# The Dental College

**Published:** 1862-01

**Authors:** 


### Correspondence.

MESSERS. EDITORS:—With your permission I will now, after a somewhat protracted silence, renew my correspondence, and in this letter, give you a few items, and some reflections.

*The Dental College* located here is in a prosperous condition, having a class of *forty-four* students—of more than ordinary intelligence in appearance—which, considering the "times," is very encouraging.

This institution has now become the leading school in our profession; the locality and the ability of its faculty fully warranting such a position.

